# A novel statistical framework for meta-analysis of total mediation effect with high-dimensional omics mediators in large-scale genomic consortia

**DOI:** 10.1371/journal.pgen.1011483

**Published:** 2024-11-19

**Authors:** Zhichao Xu, Peng Wei

**Affiliations:** Department of Biostatistics, The University of Texas MD Anderson Cancer Center, Houston, Texas, United States of America; University of Michigan, UNITED STATES OF AMERICA

## Abstract

Meta-analysis is used to aggregate the effects of interest across multiple studies, while its methodology is largely underexplored in mediation analysis, particularly in estimating the total mediation effect of high-dimensional omics mediators. Large-scale genomic consortia, such as the Trans-Omics for Precision Medicine (TOPMed) program, comprise multiple cohorts with diverse technologies to elucidate the genetic architecture and biological mechanisms underlying complex human traits and diseases. Leveraging the recent established asymptotic standard error of the R-squared (*R*^2^)-based mediation effect estimation for high-dimensional omics mediators, we have developed a novel meta-analysis framework requiring only summary statistics and allowing inter-study heterogeneity. Whereas the proposed meta-analysis can uniquely evaluate and account for potential effect heterogeneity across studies due to, for example, varying genomic profiling platforms, our extensive simulations showed that the developed method was more computationally efficient and yielded satisfactory operating characteristics comparable to analysis of the pooled individual-level data when there was no inter-study heterogeneity. We applied the developed method to 5 TOPMed studies with over 5800 participants to estimate the mediation effects of gene expression on age-related variation in systolic blood pressure and sex-related variation in high-density lipoprotein (HDL) cholesterol. The proposed method is available in R package MetaR2M on GitHub.

## Author summary

We have developed a novel meta-analysis framework to combine the estimates of the total mediation effect of high-dimensional omics mediators on complex traits from multiple studies in large-scale genomic consortia. By applying the developed method to genome-wide gene expression data from five studies with over 5,800 participants, we were able to demonstrate that our approach is not only computationally efficient but also yields reliable results. We illustrate how certain genes and biological pathways can influence age-related changes in blood pressure and sex differences in high-density lipoprotein (HDL) cholesterol levels. Our new tool, available as an R package MetaR2M on GitHub, makes it easier for researchers to analyze such complex data. This could lead to a better understanding of the genetic architecture and biological mechanisms underlying complex human traits and diseases.

## Introduction

Large-scale genomic consortia and biobanks have facilitated genetic and genomic research by providing data and tools to probe into complex human diseases and traits with unparalleled depth and applicability [1–3]. For instance, in our motivating example, the National Heart, Lung, and Blood Institute’s (NHLBI) Trans-Omics for Precision Medicine (TOPMed) project brings together over 85 cohorts consisting of more than 180,000 participants using various high-throughput profiling technologies to elucidate the genetic architecture and biological mechanisms underlying complex human traits [4]. Advances in technology and data sharing have made individual participant data more accessible [5, 6]. However, the acquisition and analysis of such individual-level data is time-consuming, financially demanding, and limited by privacy concerns.

High-dimensional mediation analysis is a crucial analytical approach focused on evaluating the mediating role of molecular phenotypes, such as gene expression, in the relationship between environmental exposure/risk factor and health outcomes [7–12]. A variance-based R-squared measure, denoted as $R_{Med}^{2}$, was proposed to estimate the total mediation effect in the high-dimensional setting [13, 14]. A recently developed two-stage cross-fitted interval estimation procedure for $R_{Med}^{2}$ enables the implementation of meta-analysis in mediation analysis due to its availability of asymptotic standard error and computational efficiency [15], as to be pursued here.

Meta-analysis is a powerful tool for synthesizing the effects of interest across multiple similar individual studies [16, 17]. Established meta-analysis techniques use summary statistics to resolve the difficulties in accessing individual-level data [18–22]. Fixed-effects meta-analysis stands out as the most widely-used and robust method for combining findings from multiple genetic studies [23]. Fixed-effects models require the assumption that the true effects of interest are identical across all studies. Within this domain, the inverse variance weighting method is widely adopted, attributing weights to each study based on the inverse of the sampling variance of the estimator of interest, for example, estimated odds ratio for binary data [24]. The Mantel-Haenszel method computes a weighted average of odds ratios, with weights being proportional to the size and variability of each study [19]. Random-effects models are used when there is heterogeneity across the studies in the meta-analysis. The DerSimonian and Laird (DL) estimator is favored for its simplicity and robustness [18]. Several authors have highlighted the importance of including a considerable number of studies in the random-effects meta-analysis to ensure the reliability of inferential results [25, 26]. More recently, the median-unbiased Paule-Mandel (MPM) estimator has been proposed to estimate the heterogeneity from the median of the generalized *Q* statistic proposed by Cochran instead of its expected value [27, 28].

Meta-analysis and systematic reviews have been extensively applied in mediation analysis to identify potential mediators influencing health-related outcomes [29–33]. However, its methodology is largely underexplored in high-dimensional mediation analysis, particularly in estimating the total mediation effect of high-dimensional omics mediators [12]. For example, TOPMed has generated over 48,000 RNA sequencing (RNA-seq) samples across 27 participating cohorts of diverse race/ethnicity, sex and age distribution (https://nhlbi.sph.umich.edu/omics/index.php, accessed on October 16, 2024). Although the RNA-seq data were centrally generated at the Genomic Sequencing Centers, the individual-level RNA-seq data are returned to individual cohorts and typically not shared across cohorts. A working group for a specific phenotype (e.g., lipids) within the TOPMed consortium develops a common analysis plan and analysts from each participating cohort executes the analysis plan and shares the summary data for meta-analysis ([21]). To address this unmet need in the emergence of large-scale genomic profiling, we introduce a novel meta-analysis framework, allowing for both fixed-effects and random-effects, to estimate the total mediation effect in high-dimensional settings. This framework requires only summary statistics and allows between-study heterogeneity arising from factors such as differences in high-throughput technologies (microarray vs. RNA-sequencing) and diverse ethnicity. Our extensive simulations show that the efficiency and coverage probability when using summary statistics are comparable to those achieved with the individual-level data in meta-analysis. Applying this innovative framework, we conducted a meta-analysis across various cohorts from the TOPMed Framingham Heart Study (FHS) and the Multi-Ethnic Study of Atherosclerosis (MESA) to estimate the mediation effects of gene expression on age-related variation in systolic blood pressure (BP) and sex-related variation in high-density lipoprotein (HDL) cholesterol. The proposed meta-analysis framework is implemented in the R package MetaR2M available on GitHub and to be submitted to R/CRAN.

## Description of the methods

In this section, we provide the background of mediation models, potential mediators/non-mediators, and the *R*^2^-based total mediation effect. Then we review the fixed-effects and random-effects models in meta-analysis using summary statistics versus individual-level data, followed by the proposed framework for meta-analysis of total mediation effect under high-dimensional settings.

### Mediation models and $R_{Med}^{2}$ measure

**Fig 1:**
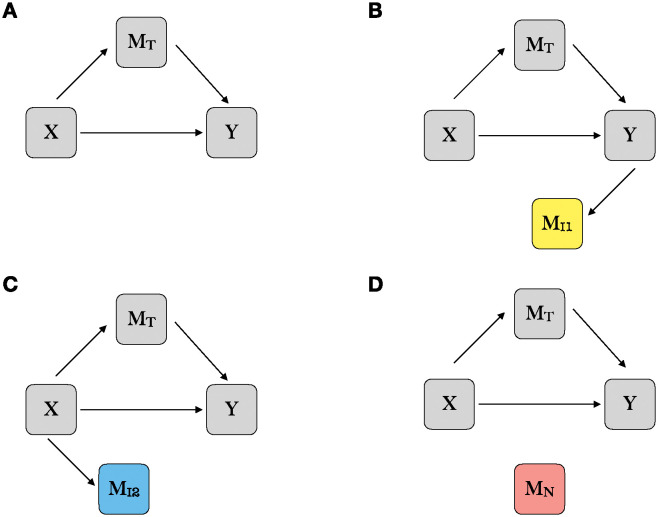
Graph representations of potential mediation models. X refers to the exposure variable. Y refers to the outcome variable. ***M***_*T*_ refers to the true mediators associated with both the exposure and the outcome. $M_{I_{1}}$ refers to the non-mediators associated with the outcome but not the exposure. $M_{I_{2}}$ refers to the non-mediators associated with the exposure but not the outcome. ***M***_*N*_ refers to the non-mediators noise variables that are not associated with either the exposure or the outcome.

Let *X* denote a *n* × 1 vector of the exposure variable, ***M*** denote a *n* × *p* matrix for *p* potential mediators, *M*_*j*_ be a *n* × 1 vector for the *j*th mediator, and *Y* represent a *n* × 1 vector of the outcome variable. Without loss of generality, we assume that all variables have been centered at 0 and scaled to have variance of 1; in addition, all measured potential confounders have been regressed out from *X*, *M*_*j*_’s and *Y* from the following equations, which constitute the mediation model:

$$\begin{array}{c} \begin{array}{cc} Y & =cX+\varepsilon_{1},\\ M_{j} & =\alpha_{j}X+\xi_{j},\\ Y & =\gamma X+M\beta +\varepsilon_{2}, \end{array} \end{array}$$ (1)

where *c*, ***α*** = (*α*_1_, …, *α*_*p*_)^*T*^, ***β*** = (*β*_1_, …, *β*_*p*_)^*T*^, and *γ* are the coefficients of regressions that can be estimated via maximum likelihood estimation (MLE), and *ε*_1_, *ε*_2_, and *ξ*_*j*_ = (*ξ*_1*j*_, …, *ξ*_*nj*_)^*T*^ are *n* × 1 vectors of random errors. Here parameter *c* is the total mediation effect linking the exposure to the outcome, and *γ* captures the direct effect of *X* on *Y* in the classical mediation analysis framework. As illustrated in Fig 1, we categorize the potential mediators $M=(M_{T},M_{I_{1}},M_{I_{2}},M_{N})$ into four groups: true mediators and three types of non-mediators [34]. True mediators $M_{T}$ (Fig 1A) are the variables associated with both the exposure and the outcome (*α*_*j*_ ≠ 0, *β* ≠ 0 for j∈T). Non-mediators $M_{I_{1}}$ (Fig 1B) are the variables associated with the outcome but not the exposure (*α*_*j*_ = 0, *β*_*j*_ ≠ 0 for $j\in I_{1}$). Similarly, non-mediators $M_{I_{2}}$ (Fig 1C) are the variables associated with the exposure but not the outcome (*α*_*j*_ ≠ 0, *β*_*j*_ = 0 for $j\in I_{2}$). Lastly, non-mediator noise variables $M_{N}$ (Fig 1D) are the variables not associated with either the exposure or the outcome (*α*_*j*_ = *β*_*j*_ = 0 for j∈N). The inclusion of the specific type of non-mediators in the high-dimensional mediation analysis could potentially bias the estimation [13].

From Eq (1), the second-moment-based total mediation effect measure $R_{Med}^{2}$ in the multiple-mediator setting is defined as follows:

$$\begin{array}{c} \begin{array}{cc} R_{Med}^{2} & =R_{YX}^{2}+R_{YM}^{2}-R_{YMX}^{2}\\ & =(1-\frac{\text{Var}(Y|X)}{\text{Var}(Y)})+(1-\frac{\text{Var}(Y|M_{T})}{\text{Var}(Y)})-(1-\frac{\text{Var}(Y|X,M_{T})}{\text{Var}(Y)})\\ & =1-\frac{\text{Var}\left(Y|X\right)+\text{Var}\left(Y|M_{T}\right)-\text{Var}\left(Y|X,M_{T}\right)}{\text{Var}(Y)}, \end{array} \end{array}$$ (2)

where $R_{YX}^{2},R_{YM}^{2}$ and $R_{YMX}^{2}$ represents the coefficient of determination for the regression models in which *Y* is regressed on $X,M_{T}$, and $\left(X,M_{T}\right)$, respectively. Next, we derive the $R_{Med}^{2}$ estimand based on Eq (1). Denote $R_{YX}^{2}={\left(cor\left(Y,X\right)\right)}^{2}$, $R_{YMX}^{2}=\text{Var}\left(\gamma X+M\beta \right)/\text{Var}\left(Y\right)$, and $R_{YM}^{2}=h^{T}V_{MM}^{-1}h$, where $h={\left(r_{M_{1}Y},r_{M_{2}Y},\cdots r_{M_{p}Y}\right)}^{T}\;\text{and}\;r_{M_{p}Y}=cor(M_{p},Y)$ is the correlation coefficient between *M*_*p*_ and *Y*, *p* is the number of mediators. *V*_*MM*_ is a *p* × *p* matrix with *cor*(*M*_*i*_, *M*_*j*_) as the (*i*, *j*)^*th*^ component. If we assume that $\xi =\left(\xi_{1},\xi_{2},\cdots ,\xi_{p}\right)∼N\left(0,D_{p\times p}\right)$ and *ε*_2_ ∼ *N*(0, *ϕ*_1_), we can show that

$$\begin{array}{c} R_{YMX}^{2}=\frac{{\left(\gamma +\beta^{T}\alpha \right)}^{2}+\beta^{T}D\beta }{\text{Var}(Y)}, \end{array}$$ (3)

$$\begin{array}{c} R_{YX}^{2}=\frac{{\left(\gamma +\beta^{T}\alpha \right)}^{2}}{\text{Var}(Y)}, \end{array}$$ (4)

$$\begin{array}{c} R_{YM}^{2}=\frac{{\left(\gamma +\beta^{T}\alpha \right)}^{2}-\gamma^{2}/\left(1+\alpha^{T}D^{-1}\alpha \right)+\beta^{T}D\beta }{\text{Var}(Y)}, \end{array}$$ (5)

$$\begin{array}{c} R_{Med}^{2}=\frac{{\left(\gamma +\beta^{T}\alpha \right)}^{2}-\gamma^{2}/\left(1+\alpha^{T}D^{-1}\alpha \right)}{\text{Var}(Y)}, \end{array}$$ (6)

where $\text{Var}\left(Y\right)={\left(\gamma +\beta^{T}\alpha \right)}^{2}+\beta^{T}D\beta +\phi_{1}$. In addition, based on Eq (1), the marginal covariance between each pair of mediators *M*_*i*_ and *M*_*j*_ is *cov*(*M*_*i*_, *M*_*j*_) = *α*_*i*_*α*_*j*_ + *cov*(*ξ*_*i*_, *ξ*_*j*_) = *α*_*i*_*α*_*j*_ + *d*_*ij*_, where *d*_*ij*_ is the (*i*, *j*)^*th*^ component of ***D***_*p*×*p*_. Given *X*, the conditional covariance of *M*_*i*_ and *M*_*j*_ is *cov*(*M*_*i*_, *M*_*j*_|*X*) = *d*_*ij*_. Therefore, if ***D***_*p*×*p*_ is a diagonal matrix, all mediators (*M*_1_, …, *M*_*p*_) are conditionally independent given *X*; however, non-diagonal ***D***_*p*×*p*_ is allowed in the above $R_{Med}^{2}$ framework to accommodate conditionally correlated mediators given *X* due to, for example, residual confounding, as shown in the simulation study.

$R_{Med}^{2}$ as a measure of total mediation effect is interpreted as the amount of variation in the outcome *Y* that is explained by exposure *X* through mediators *M* [13, 14]. Note that when *X*, *ξ*_*j*_, *ε*_1_, and *ε*_2_ are independently distributed, Eq (2) remains valid if we substitute $M_{T}$ with $M_{S}$ where S is the union of the true mediators ***M***_*T*_ and the non-mediators $M_{I_{1}}$, denoted as $S=T\cup I_{1}$ [15].

Using $R_{Med}^{2}$ as a measure of the total mediation effect offers several advantages. First, $R_{Med}^{2}$ is an appealing complementary measure to traditional total mediation effect measures, such as the product measure for mediation/indirect effect $\sum_{j=1}^{p}\alpha_{j}\beta_{j}$, by avoiding the issue of cancellation from component-wise mediation effects *α*_*j*_*β*_*j*_’s of different directions [35, 36]. Second, since $R_{Med}^{2}$ is defined based on the coefficient of determination *R*^2^, it allows the mediators to be correlated which is likely the case in high-dimensional genomics settings [13]. Third, $R_{Med}^{2}$ can be extended beyond continuous outcomes, such as time-to-event outcomes, which relax the rare event assumption as required by the product measure [14].

We also consider the Shared Over Simple (SOS) measure. Defined as $SOS=R_{Med}^{2}/R_{Y,X}^{2}$, this measure represents the standardized variance in the outcome related to the exposure that intersects with the mediators [37]:

$$SOS=\frac{R_{Med}^{2}}{R_{Y,X}^{2}}=1-\frac{\gamma^{2}}{{\left(\gamma +\beta^{T}\alpha \right)}^{2}\left(1+\alpha^{T}D^{-1}\alpha \right)}.$$

The natural indirect effect (NIE) is a counterfactual-based causal mediation effect measure, expressed as NIE = ***β***^*T*^***α*** under some strong assumptions, e.g., no unmeasured confounders between (1) the exposure and the outcome, (2) the exposure and the mediators, and (3) the mediators and the outcome [38, 39]. The proportion measure is characterized as the fraction of the total effect mediated by the mediators, denoted as ***β***^*T*^***α***/(*γ* + ***β***^*T*^***α***), where *γ* is the direct effect. Therefore, we have

$${\left(1-proportion\right)}^{2}=\frac{\gamma^{2}}{{\left(\gamma +\beta^{T}\alpha \right)}^{2}}=\left(1-SOS\right)\left(1+\alpha^{T}D^{-1}\alpha \right).$$

When the SOS equals 1, the proportion mediated also equals 1. However, when ***β***^*T*^*α* = 0 (i.e., the proportion measure = 0), but some individual pathways *α*_*j*_*β*_*j*_ ≠ 0, the proportion mediated measure is unable to capture the mediation effect, while the SOS still can, as shown in our prior work ([13, 14]).

### Meta-analysis framework for $R_{Med}^{2}$ measure

Recently, a novel two-stage interval estimation procedure using cross-fitted Ordinary Least Squares (OLS) regressions, CF-OLS, for estimating $R_{Med}^{2}$ in a single study has been proposed [15]. This method is based on cross-fitting and sample-splitting techniques and is tailored for estimating the confidence interval of total mediation effect in high-dimensional mediators settings [15]. The newly derived asymptotic distribution and, thus, the standard error, of the estimator makes it possible for meta-analysis using summary statistics, i.e., point estimate and standard error of the estimated total mediation effect from each study.

In CF-OLS, following the data split into two subsamples, the initial step involves variable selection. It is worth noting that the presence of non-mediator $M_{I_{1}}$ and noise variables $M_{N}$ does not affect the estimation when all true mediators and non-mediators are independent. However, non-mediator $M_{I_{2}}$ can introduce bias and inconsistency, especially in high-dimensional settings [13]. Therefore, we used the iterative Sure Independence Screening (iSIS) [40] in conjunction with the Minimax Concave Penalty (MCP) [41] screening procedure, known as iSIS-MCP, to identify and filter out the non-mediator $M_{I_{2}}$. Subsequently, we applied the False Discovery Rate (FDR) procedure to further exclude non-mediator $M_{I_{1}}$ and noise variables $M_{N}$, as they might bias the results when they are highly correlated [15]. With the true mediators $M_{T}$ selected in each of the two subsamples, the inference of $R_{Med}^{2}$ is conducted based on the asymptotic standard error of its estimator ${\hat{R}}_{Med}^{2}$. After the variable selection procedure, we will have

$$\begin{array}{c} \begin{array}{c} M_{j}=\alpha_{j}X+\xi_{j},\;Y=\gamma X+M_{T}\beta +\varepsilon . \end{array} \end{array}$$ (7)

If certain assumptions are met and the mediator selection satisfies the sure screening property [15], then it holds that

$$\begin{array}{c} \sqrt{n}\left({\hat{R}}_{Med}^{2}-R_{Med}^{2}\right)/\sqrt{u^{\prime }Au}\overset{d}{⟶}N\left(0,1\right), \end{array}$$ (8)

where $u={\left(1/\text{Var}\left(Y\right),-1/\text{Var}\left(Y\right),-1/\text{Var}\left(Y\right),\left(\text{Var}\left(Y|X\right)+\text{Var}\left(Y|M_{T}\right)-\text{Var}\left(Y|X,M_{T}\right)\right)/\text{Var}{\left(Y\right)}^{2}\right)}^{\prime }$ and A is the (constant) covariance matrix of (*ε*^2^, *η*^2^, *ζ*^2^, *Y*^2^) [15]. Specifically,

$$\begin{array}{c} \begin{array}{cc} \eta & =\varepsilon +\{M-\text{E}(M|X)\}\beta ,\\ \zeta & =\gamma \{X-\text{E}(X|M_{T})\}+\varepsilon . \end{array} \end{array}$$ (9)

The above result indicates that ${\hat{R}}_{Med}^{2}$ is a consistent estimator of $R_{Med}^{2}$ and follows a normal distribution, based on which the standard error and a 95% confidence interval can be analytically derived. We estimate the asymptotic covariance matrix ***A*** by the residuals of the corresponding linear regressions via the OLS.

**Fig 2:**
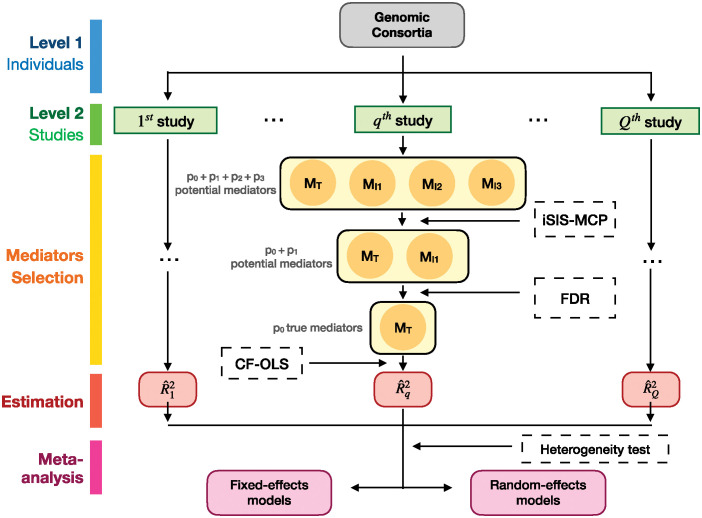
Overall workflow of meta-analysis of $R_{Med}^{2}$ in high-dimensional mediation analysis. (*p*_0_, *p*_1_, *p*_2_, *p*_3_) refers to the number of true mediators, two types of non-mediators, and noise variables $\left(M_{T},M_{I_{1}},M_{I_{2}},M_{N}\right)$, respectively.

Fig 2 illustrates the workflow of our proposed meta-analysis approach to estimating $R_{Med}^{2}$ from multiple studies under high-dimensional settings. In a large-scale genomic epidemiology consortium, we first identify the potential studies relevant to our outcome of interest. Then we apply the CL-OLS to each study independently, obtaining estimates of the *R*^2^-based mediation effect in each study.

Suppose that there are *Q* independent studies, each involving *n*_*q*_ participants, *q* = 1, 2, …*Q*. Let ${\hat{R}}_{q}^{2}$ be the estimator of $R_{Med}^{2}$ for the *q*-th study obtained using CF-OLS. Additionally, let ${\tilde{R}}^{2}$ denote the estimator of $R_{Med}^{2}$ based on the individual-level data, which pools together all the studies. The fixed-effects model in meta-analysis assumes that there is no true variability between studies beyond random sampling error. Let *R*^2^ denote the common true total mediation effect shared by all *Q* studies. The widely adopted inverse-variance estimator of $R_{Med}^{2}$ and its corresponding variance can be described as follows:

$$\begin{array}{c} \begin{array}{cc} {\hat{R}}_{IW}^{2} & =\frac{\sum_{q=1}^{Q}{\hat{R}}_{q}^{2}/\text{Var}\left({\hat{R}}_{q}^{2}\right)}{\sum_{q=1}^{Q}1/\text{Var}\left({\hat{R}}_{q}^{2}\right)},\;\text{Var}\left({\hat{R}}_{IW}^{2}\right)=\frac{1}{\sum_{q=1}^{Q}1/\text{Var}\left({\hat{R}}_{q}^{2}\right)}. \end{array} \end{array}$$ (10)

It has been shown that ${\hat{R}}_{IW}^{2}$ using summary statistics has the same asymptotic efficiency as using the individual-level data for all commonly used parametric models [42]. Consequently, $\text{Var}\left({\tilde{R}}^{2}\right)={\left(\sum_{q=1}^{Q}\text{Var}{\left({\tilde{R}}_{q}^{2}\right)}^{-1}\right)}^{-1}$, where ${\tilde{R}}^{2}$ and ${\hat{R}}_{q}^{2},q=1,2,\ldots Q$, converge to *R*^2^ under standard regularity conditions [43].

The random-effects model in meta-analysis combines data from multiple studies, accounting for both within-study and between-study variability. In contrast to the fixed-effects model, the random-effects model in meta-analysis acknowledges that variations in the true effect size among studies can arise from factors beyond random sampling error. Consider the random-effects model $R_{q}^{2}=R^{2}+\delta_{q}$, where *δ*_*q*_ ∼ *N*(0, *τ*^2^) for *q* = 1, 2, …*Q*. Let *S*_*q*_ denote the estimated variance of ${\hat{R}}_{q}^{2}$. Under the assumption that the sample size *n*_*q*_ in the *q*-th study is large enough and standard regularity conditions hold, the estimate of *R*^2^ is:

$$\begin{array}{c} \begin{array}{c} {\hat{R}}_{RE}^{2}={\left(\sum_{q=1}^{Q}{\left(S_{q}+{\hat{\tau }}^{2}\right)}^{-1}\right)}^{-1}\sum_{q=1}^{Q}(\frac{{\hat{R}}_{q}^{2}}{S_{q}+{\hat{\tau }}^{2}}), \end{array} \end{array}$$ (11)

where ${\hat{\tau }}^{2}$ is an estimate of the between-study variance *τ*^2^. For example, the commonly used DerSimonian and Laird estimator [18] of *τ*^2^ is given as

$$\begin{array}{c} \begin{array}{c} {\hat{\tau }}_{DL}^{2}=\text{max}\{\frac{\sum_{q=1}^{Q}S_{q}^{-1}\left({\hat{R}}_{q}^{2}-{\hat{R}}_{IW}^{2}\right)-\left(Q-1\right)}{\sum_{q=1}^{Q}S_{q}^{-1}-\sum_{q=1}^{Q}S_{q}^{-2}/\sum_{q=1}^{Q}S_{q}^{-1}},\;0\}. \end{array} \end{array}$$ (12)

Define

$$\begin{array}{c} T_{Gen}=\sum_{q=1}^{Q}{\left(S_{q}+\tau^{2}\right)}^{-1}{\left({\hat{R}}_{q}^{2}-{\hat{R}}_{IW}^{2}\right)}^{2}. \end{array}$$ (13)

The median-unbiased Paule-Mandel estimator ${\hat{\tau }}_{MPM}^{2}$ is given by the value of *τ*^2^ such that $T_{Gen}=\chi_{Q-1,0.5}^{2}$ (the median of a chi-square distribution with *Q* − 1 degrees of freedom).

Following this, a heterogeneity test is conducted. The *I*^2^ statistic is a widely employed metric for quantifying heterogeneity in meta-analyses. This statistic measures the proportion of total variation in study estimates attributed to authentic between-study heterogeneity, distinct from random sampling error [44]. A high *I*^2^ value (e.g., 50% to 100%) suggests high heterogeneity [45]. Based on the outcome of this assessment, we make a determination regarding the suitability of employing either the fixed-effects model or the random-effects model, guided by Cochran’s Q test [27].

## Verification and comparison by simulations

In this section, we performed extensive simulation studies to assess the performance of the proposed meta-analysis framework for $R_{Med}^{2}$ measure in high-dimensional mediation analysis. We computed coverage probability, asymptotic efficiency (i.e., standard error), bias, and empirical standard deviation of the estimator (i.e., the standard deviation of the sampling distribution of the estimator based on simulation replications). We conducted these evaluations under either fixed-effects or random-effects model, considering various high-dimensional settings to approximate real-world scenarios.

### Simulation design

Data were simulated using the model in Eq (1), and the errors therein *ε*_1_, and *ε*_2_ independently follow the standard normal distribution. Exposure variable *X* was simulated from the standard normal distribution *N*(0, 1) and coefficient *γ* in Eq 1 was set to 3. Let (*p*_0_, *p*_1_, *p*_2_, *p*_3_) denote the number of true mediators, two types of non-mediators, and non-mediator noise variables $\left(M_{T},M_{I_{1}},M_{I_{2}},M_{N}\right)$, respectively.

For the fixed-effects models, iSIS was independently applied to two subsamples within each CF-OLS procedure as depicted in Fig 2, for a total of 500 replications. As for the random-effects model, taking into account the sample size, we conducted 200 replications. The asymptotic standard error and bias were calculated as the means of their respective estimates across the two subsamples in the CF-OLS framework.

The performance of the two models was evaluated in various scenarios (A1)–(F1) and (A2)–(F2), respectively, each including different types or numbers of true mediators and non-mediators as follows. In scenarios A (A1 & A2), a substantial number of noise variables $M_{N}$ were added alongside the true mediators $M_{T}$; in scenarios B (B1 & B2) and scenarios C (C1 & C2), numerous non-mediators $M_{I_{2}}$ and $M_{I_{1}}$ were added to the true mediators, respectively. Scenarios D (D1 & D2) examined a combination of three types of non-mediators. Scenarios E and F (E1 & E2 & F1 & F2) explored cases where the true mediators were sparse amid a large number of noise variables.

The details of simulation scenarios (A)–(F) are shown as follows:

- (A) (A1)(*p*_0_, *p*_1_, *p*_2_, *p*_3_) = (150, 0, 0, 1350); (A2)(*p*_0_, *p*_1_, *p*_2_, *p*_3_) = (150, 0, 0, 4850).
- (B) (B1)(*p*_0_, *p*_1_, *p*_2_, *p*_3_) = (150, 0, 150, 1200); (B2)(*p*_0_, *p*_1_, *p*_2_, *p*_3_) = (150, 0, 150, 4700).
- (C) (C1)(*p*_0_, *p*_1_, *p*_2_, *p*_3_) = (150, 150, 0, 1200); (C2)(*p*_0_, *p*_1_, *p*_2_, *p*_3_) = (150, 150, 0, 4700).
- (D) (D1)(*p*_0_, *p*_1_, *p*_2_, *p*_3_) = (150, 150, 150, 1050); (D2)(*p*_0_, *p*_1_, *p*_2_, *p*_3_) = (150, 150, 150, 4550).
- (E) (E1)(*p*_0_, *p*_1_, *p*_2_, *p*_3_) = (5, 0, 0, 1495); (E2)(*p*_0_, *p*_1_, *p*_2_, *p*_3_) = (5, 0, 0, 4995).
- (F) (F1)(*p*_0_, *p*_1_, *p*_2_, *p*_3_) = (15, 0, 0, 1485); (F2)(*p*_0_, *p*_1_, *p*_2_, *p*_3_) = (15, 0, 0, 4985).

In each scenario, the same parameters ***α*** and ***β*** were simulated from a normal distribution *N*(0, 1.5^2^) across all replications, ensuring that the true $R_{Med}^{2}$ remained constant for the fixed-effects meta-analysis. In contrast, for the random-effects model, various parameters ***α*** and ***β*** were simulated from the same normal distribution *N*(0, 1.5^2^), and the true $R_{Med}^{2}$ was determined as the average value among one million sets of these parameter combinations due to the true $R_{Med}^{2}$ not being available in closed-form under the random-effects model. Independent variable *X* was sampled from a standard normal distribution *N*(0, 1), and coefficient *γ* in Eq (1) was set to 3. In scenarios (A1)–(F1), the independent and correlated putative mediators were considered. For independent putative mediators, the error *ξ*_*j*_ independently follows the standard normal distribution. For the putative correlated mediators, for any *i* = 1, …, *n* we consider (*ξ*_*i*1_, …, *ξ*_*ip*_)′ ∼ *N*(**0**_*p*×1_, ***I***_*p*_ + **Σ**) where **Σ**_*ij*_’s are iid samples from *N*(0, 0.1^2^) for 1 ≤ *i* ≠ *j* ≤ *p*_0_ + *p*_1_ and **Σ**_*ij*_ = 0 elsewhere. Let (*p*_0_, *p*_1_, *p*_2_, *p*_3_) denote the number of true mediators, two types of non-mediators, and noise variables $\left(M_{T},M_{I_{1}},M_{I_{2}},M_{N}\right)$, respectively.

The total number of variables in ***M*** was set to *p* = 1500 for the fixed-effects model and the random-effects model in scenarios (A1)–(F1) and *p* = 5000 in scenarios (A2)–(F2). The residuals *ε*_2_ were simulated from a normal distribution *N*(0, 1). Additionally, we considered *ε*_2_ generated from a *χ*^2^(2) distribution to resemble heavily skewed data often encountered in practice (Table B in S1 Text).

The number of studies for the fixed-effects model *Q*_*fixed*_ was selected from 1 (pooled original data) to 5, while for the random-effects model, *Q*_*random*_ was set to 5, 8, 10, 16, and 20. For the fixed-effects model, we also considered an uneven allocation of sample sizes for multiple studies (i.e., 750, 750, and 1500 for three studies), which mimics a more realistic scenario in practice. We initially generated data with *N* = 3000 and subsequently distributed them randomly across *Q*_*fixed*_ studies. In the case of the random-effects meta-analysis, we generated data with varying sample sizes for different *Q*_*random*_. We controlled the FDR level at 20% following the iSIS as shown in Fig 2.

### Simulation results

Table 1 presents the simulation results for the fixed-effects meta-analysis of $R_{Med}^{2}$ in a high-dimensional setting. Overall, the fixed-effects model demonstrated good performance across all scenarios when compared to the results obtained from the original individual-level data (*Q* = 1).

**Table 1:** Simulation results using the fixed-effects model for scenarios (A1)–(F1). *N* refers to the sample size for each study. CP refers to the empirical coverage probability of 95% confidence intervals based on 200 replications. *Q*_*fixed*_ refers to the number of studies. SE refers to the average asymptotic standard error. SD refers to the empirical standard deviation of replicated estimations (ground truth). The true value of $R_{Med}^{2}$ is shown in parentheses.

Scenario	*Q*_*fixed*_	*N*	Independent mediators				Correlated mediators			
			CP	Bias	SE	SD	CP	Bias	SE	SD
			%	×10^−2^	×10^−2^	×10^−2^	%	×10^−2^	×10^−2^	×10^−2^
A1 (0.867)	1	3000	96.5	-0.053	0.455	0.436	92.5	0.139	0.437	0.478
	2	1000 / 2000	96.5	-0.019	0.453	0.440	92.5	-0.055	0.423	0.458
	2	1500 / 1500	96.5	-0.020	0.453	0.440	91.0	0.152	0.433	0.465
	3	750 / 750 / 1500	95.5	0.009	0.452	0.441	92.5	0.138	0.517	0.529
	3	1000 / 1000 / 1000	96.5	0.011	0.452	0.440	93.0	0.137	0.421	0.411
	4	750 / 750 / 750 / 750	96.0	0.042	0.451	0.444	92.0	0.091	0.392	0.407
	5	600 / 600 / 600 / 600 / 600	95.0	0.076	0.450	0.442	90.5	0.188	0.466	0.477
B1 (0.477)	1	3000	92.0	0.053	1.319	1.431	94.0	0.457	1.322	1.321
	2	1000 / 2000	91.5	0.121	1.316	1.439	94.5	-0.034	1.303	1.390
	2	1500 / 1500	92.0	0.135	1.316	1.431	94.0	-0.415	1.211	1.213
	3	750 / 750 / 1500	92.0	0.190	1.313	1.444	92.0	0.062	1.319	1.420
	3	1000 / 1000 / 1000	91.0	0.180	1.313	1.448	93.0	-0.209	1.313	1.418
	4	750 / 750 / 750 / 750	92.0	0.232	1.311	1.441	89.0	-1.134	1.322	1.205
	5	600 / 600 / 600 / 600 / 600	91.0	0.292	1.310	1.458	92.0	-0.032	1.322	1.487
C1 (0.757)	1	3000	94.5	-0.060	0.772	0.785	93.5	0.222	0.822	0.773
	2	1000 / 2000	95.0	-0.022	0.770	0.789	93.0	0.160	0.743	0.766
	2	1500 / 1500	94.5	-0.020	0.770	0.782	94.0	0.079	0.719	0.783
	3	750 / 750 / 1500	94.0	0.025	0.769	0.785	89.5	-0.249	0.838	0.939
	3	1000 / 1000 / 1000	94.5	0.023	0.769	0.795	88.0	0.468	0.736	0.747
	4	750 / 750 / 750 / 750	94.5	0.071	0.767	0.789	94.5	-0.030	0.861	0.900
	5	600 / 600 / 600 / 600 / 600	94.0	0.105	0.766	0.797	95.0	-0.002	0.760	0.834
D1 (0.322)	1	3000	93.5	-0.009	1.403	1.476	96.5	0.358	1.402	1.301
	2	1000 / 2000	93.5	0.019	1.402	1.480	92.0	-0.064	1.403	1.497
	2	1500 / 1500	94.0	0.027	1.402	1.468	93.5	-0.380	1.407	1.422
	3	750 / 750 / 1500	93.0	0.046	1.402	1.498	94.5	0.120	1.401	1.437
	3	1000 / 1000 / 1000	92.5	0.055	1.400	1.500	94.0	-0.295	1.403	1.397
	4	750 / 750 / 750 / 750	92.0	0.059	1.401	1.511	95.0	-0.258	1.407	1.327
	5	600 / 600 / 600 / 600 / 600	93.0	0.115	1.400	1.510	95.0	-0.245	1.396	1.393
E1 (0.546)	1	3000	93.5	-0.167	1.213	1.237	96.0	-0.153	1.196	1.197
	2	1000 / 2000	94.0	-0.098	1.210	1.235	93.0	0.336	1.218	1.243
	2	1500 / 1500	94.0	-0.090	1.210	1.236	91.0	0.639	1.175	1.176
	3	750 / 750 / 1500	93.5	-0.029	1.207	1.226	94.5	0.009	1.174	1.233
	3	1000 / 1000 / 1000	94.0	-0.032	1.207	1.226	92.5	0.563	1.253	1.315
	4	750 / 750 / 750 / 750	94.0	0.037	1.204	1.212	93.5	0.045	1.214	1.227
	5	600 / 600 / 600 / 600 / 600	93.5	0.098	1.201	1.233	93.5	-0.367	1.141	1.177
F1 (0.510)	1	3000	95.0	-0.227	1.280	1.264	93.5	-0.151	1.265	1.355
	2	1000 / 2000	95.0	-0.124	1.277	1.272	93.5	-0.012	1.285	1.279
	2	1500 / 1500	95.0	-0.109	1.277	1.270	92.5	0.185	1.272	1.304
	3	750 / 750 / 1500	95.5	-0.036	1.273	1.272	93.0	0.318	1.303	1.304
	3	1000 / 1000 / 1000	95.0	-0.031	1.274	1.267	95.0	0.247	1.276	1.311
	4	750 / 750 / 750 / 750	95.0	0.045	1.270	1.270	93.5	-0.185	1.264	1.326
	5	600 / 600 / 600 / 600 / 600	95.0	0.111	1.266	1.288	92.0	0.218	1.217	1.304

The empirical coverage probability of the fixed-effects model remained consistently satisfactory across all scenarios with independent mediators, closely approximating the nominal 95% level. Even in scenario (D1), where all three types of non-mediators were included and the sample sizes were down to 600 across the *Q* = 5 studies, the coverage probability remained above 90%. The coverage probability with correlated putative mediators generally performed reasonably well, maintaining above 90% except for some cases in scenarios (B1) and (C1). The observed bias was lowest in scenarios (B1) and (D1) when using the original data for the independent mediators, but this was not the case in other scenarios. In addition, the asymptotic standard errors (SEs) approximated the simulation-based standard deviations (SDs) of the estimator well, the latter of which was considered as the ground truth. A similar conclusion was reached when we included a substantial number of noise mediators $M_{N}$ in scenarios (A2) through (F2), as detailed in the Table A in S1 Text.

Tables 2 and 3 summarize the results based on two different between-study variance estimators in the random-effects meta-analysis of $R_{Med}^{2}$ in high-dimensional settings. The coverage probability demonstrated satisfactory results when *Q*_*random*_ had a moderate value. However, for *Q*_*random*_ = 5, the coverage probability fell below 90% using the DerSimonian-Laird (DL) estimator. The empirical coverage probability of random effects models, utilizing the median-unbiased Paule-Mandel (MPM) estimator, remained consistently satisfactory across nearly all scenarios involving both independent and correlated mediators. Previous studies have indicated that achieving the correct coverage probability in random-effects meta-analysis may require *Q* = 50 studies [46]. Comparing the DL estimator and the MPM estimator, we observed similar bias and asymptotic standard errors. Notably, the MPM estimator tended to outperform the DL estimator in terms of coverage probability and approximation of the asymptotic SE to the simulation-based SD (ground truth), especially when the number of studies was limited (*Q*_*random*_ ≤ 10).

**Table 2:** Simulation results using the random-effects model with independent mediators for scenarios (A1)–(F1). *N* refers to the sample size for each study. CP refers to coverage probability based on 200 replications. *Q*_*random*_ refers to the number of studies. SE refers to the average asymptotic standard error. SD refers to the empirical standard deviation of replicated estimations (ground truth). The true value of $R_{Med}^{2}$ is shown in parentheses.

Scenario	*Q*_*random*_	*N*	DerSimonian-Laird				Median-unbiased Paule-Mandel			
			CP	Bias	SE	SD	CP	Bias	SE	SD
			%	×10^−2^	×10^−2^	×10^−2^	%	×10^−2^	×10^−2^	×10^−2^
A1 (0.468)	5	2400	88.0	1.284	14.939	13.172	92.0	1.278	14.641	13.156
	8	1500	91.5	1.285	12.130	9.878	94.0	1.280	11.139	9.865
	10	1200	92.0	0.908	11.392	8.950	94.0	0.904	9.908	8.936
	16	750	93.5	0.482	9.118	7.748	94.5	0.483	7.729	7.741
	20	600	93.5	0.694	8.262	6.772	94.5	0.698	6.897	6.767
B1 (0.489)	5	2400	80.5	-1.291	13.919	12.948	88.0	-1.294	13.827	12.938
	8	1500	90.0	-1.734	12.618	9.844	92.0	-1.738	10.907	9.833
	10	1200	94.0	-1.812	11.667	8.358	96.5	-1.814	9.798	8.349
	16	750	97.0	-1.657	9.496	6.971	95.5	-1.654	7.680	6.967
	20	600	95.0	-1.552	8.411	6.756	92.5	-1.546	6.833	6.754
C1 (0.357)	5	2400	85.5	1.248	13.170	12.135	92.5	1.257	13.072	12.116
	8	1500	90.0	1.172	10.928	8.928	95.0	1.176	10.044	8.919
	10	1200	93.5	0.836	10.015	8.100	94.5	0.840	8.947	8.092
	16	750	94.0	0.473	8.259	6.934	95.0	0.478	6.972	6.928
	20	600	95.0	0.625	7.479	6.126	95.5	0.628	6.214	6.125
D1 (0.380)	5	2400	85.0	-1.781	12.399	11.716	90.5	-1.771	12.334	11.698
	8	1500	88.5	-2.082	10.777	8.813	92.5	-2.076	9.746	8.802
	10	1200	91.5	-2.103	10.011	7.566	94.0	-2.097	8.770	7.557
	16	750	92.5	-1.923	8.101	6.410	94.0	-1.919	6.857	6.407
	20	600	95.0	-1.682	7.475	6.162	92.0	-1.681	6.136	6.166
E1 (0.494)	5	2400	84.5	-3.406	14.235	15.673	89.0	-3.421	15.865	15.655
	8	1500	87.5	-3.389	12.057	12.478	89.0	-3.408	12.591	12.451
	10	1200	90.5	-3.567	10.892	11.623	89.0	-3.588	11.219	11.592
	16	750	92.5	-3.923	10.101	9.410	92.0	-4.919	9.857	9.407
	20	600	91.0	-4.132	7.859	8.456	88.5	-4.154	7.950	8.425
F1 (0.481)	5	2400	82.5	-0.829	14.809	13.561	86.5	-0.834	14.718	13.547
	8	1500	87.5	-0.355	12.959	11.070	92.5	-0.360	11.484	11.054
	10	1200	88.5	-0.011	11.915	9.888	91.5	-0.016	10.147	9.876
	16	750	95.0	-0.453	9.848	7.698	93.5	-0.450	7.867	7.692
	20	600	96.0	-0.227	8.904	6.736	92.5	-0.221	6.992	6.733

**Table 3:** Simulation results using the random-effects model with correlated mediators for scenarios (A1)–(F1). *N* refers to the sample size for each study. CP refers to coverage probability based on 200 replications. *Q*_*random*_ refers to the number of studies. SE refers to the average asymptotic standard error. SD refers to the empirical standard deviation of replicated estimations (ground truth). The true value of $R_{Med}^{2}$ is shown in parentheses.

Scenario	*Q*_*random*_	*N*	DerSimonian-Laird				Median-unbiased Paule-Mandel			
			CP	Bias	SE	SD	CP	Bias	SE	SD
			%	×10^−2^	×10^−2^	×10^−2^	%	×10^−2^	×10^−2^	×10^−2^
A1 (0.469)	5	2400	88.0	1.125	15.023	13.160	91.0	1.120	14.654	13.145
	8	1500	92.0	1.231	12.109	9.779	93.5	1.226	11.160	9.765
	10	1200	90.0	0.987	11.261	9.036	94.0	0.983	9.907	9.023
	16	750	94.0	0.492	9.378	7.711	94.0	0.495	7.754	7.703
	20	600	93.0	0.504	8.405	6.866	95.0	0.508	6.935	6.859
B1 (0.490)	5	2400	83.0	-1.251	14.050	12.983	89.5	-1.255	13.886	12.973
	8	1500	90.0	-1.766	12.653	9.844	91.5	-1.770	10.895	9.832
	10	1200	93.5	-1.845	11.693	8.323	96.0	-1.846	9.815	8.314
	16	750	96.5	-2.076	9.612	6.987	95.0	-2.073	7.717	6.982
	20	600	95.5	-1.790	8.794	6.741	92.5	-1.782	6.867	6.742
C1 (0.358)	5	2400	85.5	1.287	13.174	12.063	93.0	1.296	13.050	12.042
	8	1500	89.0	1.124	10.880	8.971	94.5	1.133	10.073	8.955
	10	1200	91.5	0.591	9.915	8.241	94.5	0.600	8.972	8.224
	16	750	93.5	0.377	8.205	6.888	95.5	0.385	7.017	6.877
	20	600	95.0	0.323	7.473	6.205	95.0	0.327	6.236	6.201
D1 (0.381)	5	2400	85.5	-1.849	12.552	11.677	90.0	-1.841	12.405	11.661
	8	1500	88.0	-2.199	10.705	8.927	92.5	-2.186	9.755	8.905
	10	1200	91.5	-2.310	10.008	7.602	93.5	-2.303	8.803	7.593
	16	750	93.0	-2.067	8.127	6.388	94.0	-2.063	6.911	6.385
	20	600	95.5	-1.890	7.527	6.129	91.5	-1.888	6.170	6.132
E1 (0.495)	5	2400	84.5	-3.424	14.246	15.642	89.0	-3.439	15.838	15.624
	8	1500	88.5	-3.417	12.005	12.496	89.5	-3.437	12.618	12.468
	10	1200	89.0	-3.382	10.877	11.521	88.5	-3.402	11.132	11.491
	16	750	90.5	-4.123	8.802	9.174	88.0	-4.143	8.879	9.148
	20	600	92.0	-4.303	7.951	8.466	88.0	-4.323	8.004	8.438
F1 (0.481)	5	2400	81.5	-0.813	14.827	13.518	87.0	-0.817	14.703	13.504
	8	1500	87.5	-0.176	12.961	11.149	91.5	-0.179	11.482	11.131
	10	1200	89.0	0.024	11.839	9.891	92.0	0.018	10.131	9.878
	16	750	94.5	-0.307	9.916	7.657	92.5	-0.304	7.880	7.649
	20	600	96.5	-0.244	8.914	6.758	92.5	-0.237	6.985	6.755

## Real data applications

In this section, we describe the cohorts in the TOPMed program, including subject recruitment, ethnic diversity, and high-throughput technologies. We then apply our proposed meta-analysis framework to cardiovascular disease (CVD) traits in these studies as a proof of concept.

### Heterogeneity in cohorts, ethnic representations, and mRNA profiling technologies

The Trans-Omics for Precision Medicine (TOPMed) program represents a groundbreaking initiative launched by the National Heart, Lung, and Blood Institute (NHLBI) with the vision of aggregating whole-genome sequencing (WGS) and other omics data from more than 85 population studies [4, 47]. The program’s objective is to uncover the genetic and molecular foundations associated with heart, lung, blood, and sleep disorders [47]. The Framingham Heart Study (FHS) began the recruitment for the Offspring cohort in 1971, which comprises the children of the Original cohort and their spouses. The Offspring cohort consists of 5,124 individuals, of which 52% are female [48]. In 2002, FHS initiated the Third-Generation cohort, encompassing the children of the Offspring cohort, consisting of 4,095 participants of which 54% are female [49]. The vast majority of the FHS participants are Non-Hispanic Whites. Another active and comprehensive cohort study from the TOPMed is the Multi-Ethnic Study of Atherosclerosis (MESA), encompassing 6,814 individuals aged 45 to 84 from six U.S. communities [50]. MESA is dedicated to unraveling the risk factors that contribute to the development of CVD, particularly focusing on atherosclerosis, across 4 ethnic groups, including Non-Hispanic Whites, African Americans, Hispanics, and Chinese Americans [51].

The transcriptome encompasses all messenger RNAs (mRNAs)/transcripts in a cell during a specific stage or condition. It is crucial for deciphering the genome’s functional elements, understanding cellular components, and gaining insights into development and disease [52]. Typically, hybridization-based microarray gene expression profiling is cost-effective and high-throughput but depends on current genomic knowledge [53]. Conversely, RNA sequencing (RNA-seq) facilitates the identification of new gene transcripts and non-coding RNAs [54]. Thanks to the decreasing cost of next-generation sequencing technologies, RNA-seq has become more affordable and feasible in large-scale studies such as the FHS and MESA.

### Applications to CVD traits

Hypertension stands as the primary contributor to global CVD and premature mortality [55]. In 2010, 31.1% of the global adult population (1.39 billion), were diagnosed with hypertension, characterized by a systolic blood pressure (BP) of ≥140 mmHg and/or a diastolic BP of ≥90 mmHg [56]. Parallel to the observed increase in hypertension prevalence, the estimated counts of all-cause and CVD mortalities associated with high BP showed a significant rise from 1990 to 2015 [57]. On the other hand, previous epidemiological studies consistently identified inverse linear associations between high-density lipoprotein cholesterol (HDL-C) levels and the risks associated with CVD and mortality [58–60]. Meanwhile, many findings have highlighted notable sexual dimorphism in HDL-C levels and functionality [61, 62].

We applied our developed meta-analysis method to the FHS Offspring cohort, the FHS Third-Generation cohort, and the MESA cohort to estimate the mediation effects of gene expression on age-related variation in systolic BP and sex-related variation in HDL-C. Systolic BP was determined by averaging two physician-taken readings (rounded to the nearest 2 mm Hg). For individuals on anti-hypertensive medication, an adjustment was made by adding 15 mm Hg to their reading [63]. HDL-C was measured from EDTA plasma (in mg/dL), and age was recorded based on the participant’s age at the time of examination. The covariates included body mass index (BMI, expressed in *kg*/*m*^2^), dichotomized smoking status (current smoker or non-smoker), and dichotomized drinking status (never or ever).

We also included the top 10 principal components (PCs) of genome-wide gene expression data, selected based on eigenvalues, as covariates in the mediation analysis models. The use of PCs is common in genome-wide association studies, where they play a key role in correcting for subtle population stratification and controlling for confounding genetic backgrounds [64]. We have recently shown that adjusting for the top PCs as covariates in high-dimensional mediation analysis can effectively reduce the conditional correlations among the mediators given *X*, and, thus, mitigate unmeasured confounding effects ([15]). For the MESA cohort, we additionally adjusted for race/ethnicity, whereas for the FHS cohorts, all subjects are White. This highlights the advantage of our proposed meta-analysis approach in addressing heterogeneity in covariates across different cohorts.

When a variable, either age or sex, was considered the exposure of interest, the other was incorporated as a covariate in the model by regressing out the covariate and working on the residuals subsequently [15]. In the FHS Offspring and Third-Generation cohorts, expression profiling for 17,873 genes/transcripts was conducted using the Affymetrix Human Exon 1.0 ST GeneChip, derived from whole blood mRNA [65]. On the other hand, as part of the TOPMed program, RNA-seq was performed on whole blood in these FHS cohorts using the Illumina NovaSeq system profiling expression of over 40,000 transcripts [66]. In the meta analysis, we only included non-overlapping participants between the microarray and RNA-seq platforms. As the sample size *n* in each cohort ranged from ∼700 to less than 2,000 and the number of transcript *p* ranged from ∼17,000 to ∼47,000, we found that the default maximum number of variables selected by iSIS (i.e., *n*/ log(*n*) [40]) as implemented in R package SIS could be too small. Instead, we used max(*n*/ log(*n*), 0.02*p) as the maximum number of variables selected by iSIS in simulations and real data applications.

Exposures, covariates, and gene expression levels were extracted from the FHS Offspring cohort’s 8^*th*^ examination, the FHS Third-Generation cohort’s 2^*nd*^ examination, and the MESA cohort’s 1^*st*^ examination. Phenotype data was gathered from the Offspring cohort’s 9^*th*^ examination, the Third-Generation cohort’s 3^*rd*^ examination, and the MESA cohort’s 1^*st*^ examination to ensure temporal order that the exposure affects the mediators which in turn precedes the outcome [67]. We also pooled all FHS cohorts and the MESA cohort into a single dataset to conduct a MEGA-analysis for comparison with the meta-analysis. For the gene expression data, we used the overlapping transcripts across all five cohorts as putative mediators, resulting in the loss of a large number of transcripts (Table E in S1 Text).

**Fig 3:**
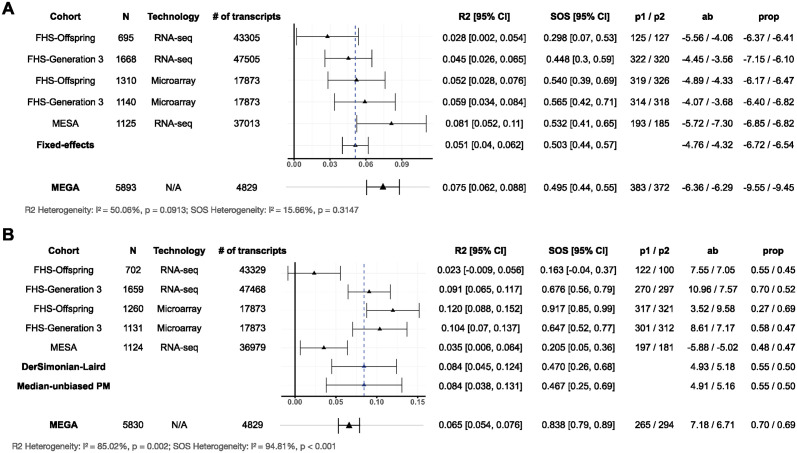
Meta-analysis results using the CF-OLS in 8 different cohorts from the NHLBI TOPMed program. (A) Fixed-effects model results of mediation effect of gene expression between age and systolic BP and (B) random-effects model results of mediation effect of gene expression between sex and HDL-C. **N** refers to the sample size. Technology refers to the high-throughput gene expression profiling technology. # of transcripts refers to the number of genes measured from the gene expression profiling. p1 / p2 refers to the number of transcripts selected in the first and second subsample, respectively. R2 refers to the total mediation effect $R_{Med}^{2}$. CI refers to the confidence interval. CA refers to the Chinese American. AA refers to African American. **ab** refers to the product measure in the first and second subsample. **prop** refers to the proportion measure in the first and second subsample.

In Fig 3A, we present fixed-effects meta-analysis results investigating the total mediation effect of gene expression in the relationship between age and systolic BP across 5 cohorts from the TOPMed program. Both the sample size and the number of profiled transcripts varied across cohorts. We employed the CF-OLS on each cohort to identify the true mediators, subsequently obtaining the $R_{Med}^{2}$ estimate along with its 95% confidence interval.

Given the Eq 1, the product measure is defined as ***β***^*T*^***α***. The total effect measure is given by $\sum_{j=1}^{p}\alpha_{j}\beta_{j}+\gamma$. The meta-analysis of these mean-based measures was conducted using standard errors calculated from 200 bootstrap resamplings within each cohort, which entails significantly more computational burden compared to our proposed meta-analysis framework. For example, in the FHS Third-Generation RNA-seq cohort, a total of 1,668 subjects with complete data were included in the analysis. We applied the CF-OLS procedure to perform variable selection out of the 47,505 transcripts measured using RNA-seq, in which 322 and 320 transcripts remained in each of the two subsamples for the estimation. We then estimated that 4.5% (95% CI = (2.6%, 6.5%)) of systolic BP variation was attributable to the indirect effect of age, mediated by gene expression, i.e., SOS = 44.8% (95% CI = (30.2%, 59.3%)) of the age-related variation in systolic BP was mediated by gene expression in the FHS Third-Generation RNA-seq cohort. Furthermore, we computed the *I*^2^ statistic to quantify the extent of heterogeneity, offering a measure of the degree of inconsistency in results across cohorts [45]. Given the lack of heterogeneity (*I*^2^ = 50.06%, *p* = 0.09), we opted for the proposed fixed-effects model to combine the total mediation effects from diverse cohorts. Consequently, 5.1% (95% CI = (4.0%, 6.2%)) of the variance in systolic BP was explained by age through gene expression (SOS = 50.3% (95% CI = (43.6%, 57.0%))).

Fixed-effects meta-analysis yielded comparable point estimation and confidence intervals as the previous study, suggesting that the new method effectively gives and combines reliable estimates across diverse cohorts [15].

Fig 3B displays the results of mediation analysis of gene expression in the relationship between sex and HDL-C, including the same 5 cohorts from the TOPMed program. With the observed heterogeneity between cohorts (*I*^2^ = 85.02%, *p* < 0.01), we chose the random-effects meta-analysis for $R_{Med}^{2}$. For example, the FHS Third-Generation RNA-seq cohort exhibited a total mediation effect of 9.1% (95% CI = (6.5%, 11.7%)), which is nearly four times greater than the 2.3% (95% CI = (-0.9%, 5.6%)) observed in the FHS Offspring cohort. Using the proposed random-effects meta-analysis model, we estimated that 9.4% (95% CI = (4.5%, 12.4%)) of HDL-C variation using the DL estimator could be explained by sex through the mediation of gene expression (SOS = 47.0% (95% CI = (26.0%, 69.0%))).

The MPM estimator, shown to have an edge over the DL estimator when the number of studies was limited (Table 3), was employed to estimate the between-study variance [68]. The results were comparable.

However, for systolic BP, the indirect and total effects had opposite directions. This resulted in a negative value for the proportion measure across all 5 cohorts, which is counterintuitive and difficult to interpret.

Since the sample size in each of four MESA race/ethnicity-groups was less than 500, we combined them into a single cohort (*N* = 1125 for systolic BP outcome and *N* = 1124 for HDL-C outcome) and then conducted the analysis.

We also analyzed the MESA study as four separate race/ethnicity cohorts, as detailed in Fig A in S1 Text. Given the observed lack of heterogeneity between cohorts for the systolic BP outcome (*I*^2^ = 12.72%, *p* = 0.3308), the fixed-effects model similarly concluded that 4.2% (95% CI = (3.2%, 5.3%)) of the variance in systolic BP could be explained by age through gene expression. For HDL-C, the DL estimator indicated that 6.8% (95% CI = (4.0%, 9.6%)) of the variation could be explained by sex through gene expression, with the MPM estimator providing a nearly identical estimate of 6.7% (95% CI = (3.7%, 9.8%)).

To investigate the biological pathways involved, we conducted a pathway enrichment analysis on the mediator genes selected in each cohort. We then carried out a meta-analysis of the enrichment p-values for the pathways (Table C and Table D in S1 Text). This analysis utilized the sample size-weighted Stouffer’s combination of p-values [69]. We observed that there were more enriched pathways (meta-analysis p-value ≤ 0.05) for HDL-C than for SBP. Notably, the endocytosis pathway plays a critical role in cellular processes and could influence lipid metabolism, including HDL-C levels [70].

Finally, in the MEGA analysis, we adjusted for BMI, sex or age (depending on the primary exposure of interest), smoking status, drinking status, race/ethnicity, gene expression profiling platform (microarray or RNA-seq), source cohort, and the top 10 PCs of pooled gene expression data as covariates. As shown in Fig 3, the MEGA analysis led to quite different point estimates and 95% CIs for the total mediation effects from those in the meta-analysis due to several reasons. First, the MEGA analysis imposes a common mediation model across cohorts and may introduce bias when there is heterogeneity in mediation effects. Second, a common set of genes/mediators need to be considered, leading to substantial loss of genes after intersecting the microarray and RNA-seq platforms across the 5 cohorts (Table E in S1 Text). Third, simply including gene expression profiling platform as a covariate may not adequately take into account the vast difference between microarray and RNA-seq, introducing potential bias. Last, but not the least, it cannot adjust for cohort-specific top PCs of genome-wide gene expression as covariates to mitigate cohort-specific unmeasured confounding effects.

## Discussion

We have introduced a novel and efficient method for conducting fixed-effects and random-effects meta-analyses for the $R_{Med}^{2}$ total mediation effect in high-dimensional settings. This method only requires summary statistics and accounts for between-study heterogeneity. Our approach incorporates iSIS-MCP into two subsamples to eliminate the non-mediators $M_{I_{2}}$. We then apply FDR control to filter out the non-mediators $M_{I_{1}}$ and noise variable $M_{N}$. We then obtain the point estimate and asymptotic standard error of $R_{Med}^{2}$ via the CF-OLS procedure. Depending on the results of the heterogeneity test, we subsequently perform either fixed-effects or random-effects meta-analysis.

Based on our simulations, we demonstrate that the relative efficiency and coverage probability achieved using summary statistics are comparable with those obtained from the original individual-level data in a fixed-effects meta-analysis. Additionally, our simulations indicate that conducting a meta-analysis for total mediation effect is reliable with a minimum sample size of around 300 in each study. This is particularly applicable when the study sizes are comparable, or when there are larger studies that can offset those with more limited sample sizes. Furthermore, in the more realistic scenario where the assumption of a common effect size across all studies no longer holds, the random-effects model maintains an acceptable coverage probability when the number of studies is relatively large, for example, larger than 10, which holds for most large-scale genomic consortia, such as the TOPMed program with over 85 studies and The Global Lipids Genetics Consortium with over 200 studies [71].

In the TOPMed program, as a proof of concept we applied our proposed new meta-analysis framework across various FHS and MESA cohorts to assess the mediation effects of gene expression on age-related variation in systolic BP and sex-related variation in HDL-C. Our findings closely align with results derived from the original individual-level data with much less computational cost, highlighting the efficiency of our method in handling the computational burden caused by large-scale studies. This is particularly applicable to mediation analysis in large-scale biobanks, such as the UK Biobank of over a half million participants with diverse ethnicity and multi-omics profiling based on different platforms. Meta-analysis can be an appealing alternative to analysis of the entire dataset at once in terms of computational feasibility [21] and evaluating potential heterogeneity across risk factors for common diseases and genomic profiling technologies, as demonstrated in our application to the FHS and MESA cohorts.

The $R_{Med}^{2}$ measure can not only characterize the overall mediation effect, but is also applicable to individual significant mediators. As defined in [72], the $R_{Med}^{2}$ measure for a single mediator *M*_*j*_, *j* = 1, …, *p* is: $R_{Med,j}^{2}=R_{YX}^{2}+R_{YM_{j}}^{2}-R_{YM_{j}X}^{2}$, where $R_{YM_{j}}^{2}$ and $R_{YM_{j}X}^{2}$ are from linear regression models *Y* ∼ *M*_*j*_ and *Y* ∼ *M*_*j*_ + *X*, respectively. However, in the case of multiple mediators, the remaining selected mediators *M*_(−*j*)_’s are also associated with *X* and *Y*, and possibly with *M*_*j*_. Therefore, *M*_(−*j*)_’s are exposure-outcome confounders and possibly exposure-mediator and mediator-outcome confounders [38]. To adjust for covariates and confounders, we have proposed modifying the $R_{Med}^{2}$ measure based on the partial *R*^2^ ([13] [14]): $R_{Med,j|Z}^{2}=R_{YX|Z}^{2}+R_{YM_{j}|Z}^{2}-R_{YM_{j}X|Z}^{2}$, where *Z* is the set of measured covariates and confounders including *M*_(−*j*)_’s. Conceptually, the modified $R_{Med,j|Z}^{2}$ measures the mediating effect of *M*_*j*_ conditional on *Z*.

As a proof of concept, we have applied the $R_{Med}^{2}$ and product measures to a single gene (ABCG1) that was selected in 4 out of the 5 cohorts (Fig B and Fig C, Table F in S1 Text). As shown in Fig B and Fig C in S1 Text, there was zero selected gene (mediator) that was common to all 5 cohorts in either age-SBP or sex-HDL application, highlighting the heterogeneity in high-dimensional mediator selection and challenges in single-gene-based meta-analysis of mediation effects (Table F in S1 Text). On the other hand, as shown in our real data applications (Fig 3), the total mediation effects captured by the $R_{Med}^{2}$ measure were more consistent across cohorts despite different sets of genes were selected in each cohort, highlighting the consistent biological mechanisms revealed at the transcriptomic and biological pathway levels, but not necessarily at the individual gene level.

There are some similarities between our proposed $R_{Med}^{2}$-based mediation analysis and estimating chip-heritability in genome-wide association studies (GWAS) [73]. First, both consider the phenotypic variance that can be explained by a set of single nucleotide polymorphisms (SNPs) or mediators, and, thus, avoid cancelling out of positive and negative individual SNP/mediator effects. Second, both can be estimated in the mixed-model framework by considering individual SNP’s or mediator’s effects as random [13]. However, there are some key differences between estimating heritability and our proposal in the context of mediation analysis. First, in the former, only a single model *Y* ∼ GWAS SNPs is entailed, while, in the latter, three models are needed, *Y* ∼ *X*, *Y* ∼ *M*, and *Y* ∼ *M* + *X*, where *X* is the exposure of interest and *M* are high-dimensional omics data. Including one type of non-mediators ($M_{I_{2}}$) can lead to substantial upward bias in estimating the total mediation effect. As we demonstrated both analytically and numerically [13], variable selection regarding $M_{I_{2}}$ has to be considered in the context of mediation analysis. Second, univariate summary statistics-based heritability estimation (e.g., LD score regression [74]) relies on accurate linkage disequilibrium (LD) (correlation) information among all SNPs from large external reference panels, such as those based on the 1000 Genomes and the TOPMed WGS data. On the other hand, unlike correlations/LDs among SNPs which are stable within a population (e.g., individuals of European ancestry), gene expression is highly variable in response to environmental and endogenous stimuli, leading to the lack of reliable and transferable correlations among genome-wide gene expression from external sources. This, along with the need for variable selection within each cohort, makes univariate summary statistics-based mediation analysis and its meta-analysis much more challenging than heritability estimation based on GWAS SNPs. This warrants future research.

In the context of high-dimensional gene expression data, confounders may be unknown or arise from various sources, potentially violating the identifiability assumptions in causal mediation analysis [75, 76]. In our real data application, we applied variable selection to exclude non-mediators and adjusted for the top PCs of genome-wide gene expression to account for unmeasured confounding effects [77] [15]. While more advanced methods are beyond the scope of this study, they are crucial for future research, including the use of Mendelian randomization to further explore causal interpretations [78].

## Supporting information

S1 Text Supplementary materials. The supplementary materials complement the main text and provide further simulation details, particularly in high-dimensional settings. In addition, more details are provided on (1) pathway enrichment analysis of selected mediators, (2) a sensitivity analysis by considering each race/ethnicity cohort in the MESA study separately, and (3) meta-analysis of a single gene.
(PDF)
