# Supplementary material for: A novel statistical framework for meta-analysis of total mediation effect with high-dimensional omics mediators in large-scale genomic consortia
Source: PLoS Genet. 2024 Nov 19;20(11):e1011483. doi: 10.1371/journal.pgen.1011483 (PMC11614268; doi:10.1371/journal.pgen.1011483)
Supplement: S1 Text — The supplementary materials complement the main text and provide further simulation details, particularly in high-dimensional settings. In addition, more details are provided on (1) pathway enrichment analysis of selected mediators, (2) a sensitivity analysis by considering each race/ethnicity cohort in the MESA study separately, and (3) meta-analysis of a single gene. (PDF) [file pgen.1011483.s001.pdf]

Journal Title Vol. , No. , pp. 1–8
doi: DOI HERE
Advance Access Publication Date: Day Month Year
Paper

Supplementary Materials S1 Text for "A novel statistical framework for meta-analysis of total mediation effect with high-dimensional omics mediators in large-scale genomic consortia"

Zhichao Xu[1] and Peng Wei[1,*]

[1]Department of Biostatistics, The University of Texas MD Anderson Cancer Center, Houston, 77030, Texas, United States of America

*pwei2@mdanderson.org

ABSTRACT

This supplementary material complements the main text and is organized into several sections. Here we provide further simulation details, particularly in high-dimensional settings, utilizing the CF-OLS method. In addition, we performed (1) pathway enrichment analysis of selected mediators for systolic blood pressure (BP) and high-density lipoprotein cholesterol (HDL-C), (2) a sensitivity analysis by considering each race/ethnicity cohort in the MESA study separately, and (3) meta-analysis of a single gene.

ADDITIONAL SIMULATIONS AND DETAILS

Table A presents the simulation results for the fixed-effects meta-analysis of R^2_{Med} in a high-dimensional setting in scenarios (A2)–(F2). Overall, the fixed-effects model demonstrated good performance across all scenarios when compared to the results obtained from the original individual-level data ($Q = 1$).

The details of simulation scenarios (A2)–(F2) are shown as follows:

- (A2)$(p_0, p_1, p_2, p_3) = (150, 0, 0, 4850)$.
- (B2)$(p_0, p_1, p_2, p_3) = (150, 0, 150, 4700)$.
- (C2)$(p_0, p_1, p_2, p_3) = (150, 150, 0, 4700)$.
- (D2)$(p_0, p_1, p_2, p_3) = (150, 150, 150, 4550)$.
- (E2)$(p_0, p_1, p_2, p_3) = (5, 0, 0, 4995)$.
- (F2)$(p_0, p_1, p_2, p_3) = (15, 150, 150, 4985)$.

© The Author 2022. Published by Oxford University Press. All rights reserved. For permissions, please e-mail: journals.permissions@oup.com

Table A. Simulation results using the fixed-effects model with independent mediators for scenarios (A2)–(F2). CP refers to the empirical coverage probability of 95% confidence intervals based on 200 replications. Q_{fixed} refers to the number of studies. SE refers to the average asymptotic standard error. SD refers to the empirical standard deviation of replicated estimations.

Scenario	Q	N	**CP** %	**Bias** $\times 10^{-2}$	**SE** $\times 10^{-2}$	**SD** $\times 10^{-2}$
A2	1	3000	94.50	0.006	0.452	0.480
(0.867)	2	1000 / 2000	94.00	0.044	0.450	0.483
	2	1500 / 1500	93.00	0.044	0.450	0.482
	3	750 / 750 / 1500	93.00	0.076	0.449	0.481
	3	1000/ 1000 / 1000	93.50	0.070	0.449	0.480
	4	750 / 750 / 750 / 750	91.00	0.103	0.448	0.484
	5	600 / 600 / 600 / 600 / 600	91.00	0.139	0.447	0.488
	6					
B2	1	3000	97.50	0.159	1.316	1.185
(0.322)	2	1000 / 2000	97.50	0.229	1.314	1.176
	2	1500 / 1500	97.00	0.218	1.314	1.195
	3	750 / 750 / 1500	97.50	0.281	1.311	1.200
	3	1000/ 1000 / 1000	97.50	0.285	1.311	1.187
	4	750 / 750 / 750 / 750	97.50	0.317	1.309	1.205
	5	600 / 600 / 600 / 600 / 600	97.50	0.362	1.308	1.199
	14					
C2	1	3000	93.50	0.058	0.768	0.768
(0.510)	2	1000 / 2000	93.00	0.107	0.766	0.774
	2	1500 / 1500	94.00	0.103	0.766	0.766
	3	750 / 750 / 1500	93.50	0.151	0.764	0.768
	3	1000/ 1000 / 1000	93.00	0.149	0.765	0.768
	4	750 / 750 / 750 / 750	92.50	0.198	0.763	0.777
	5	600 / 600 / 600 / 600 / 600	92.50	0.249	0.761	0.771
	22					
D2	1	3000	94.00	0.152	1.405	1.395
(0.546)	2	1000 / 2000	93.50	0.175	1.403	1.397
	2	1500 / 1500	93.50	0.175	1.403	1.394
	3	750 / 750 / 1500	93.50	0.198	1.403	1.398
	3	1000/ 1000 / 1000	94.00	0.199	1.402	1.413
	4	750 / 750 / 750 / 750	94.00	0.213	1.402	1.414
	5	600 / 600 / 600 / 600 / 600	93.00	0.231	1.400	1.426
	30					
E2	1	3000	96.50	0.055	1.208	1.171
(0.867)	2	1000 / 2000	95.50	0.126	1.205	1.177
	2	1500 / 1500	96.00	0.129	1.205	1.171
	3	750 / 750 / 1500	95.50	0.192	1.202	1.168
	3	1000/ 1000 / 1000	95.00	0.184	1.202	1.175
	4	750 / 750 / 750 / 750	95.50	0.246	1.199	1.173
	5	600 / 600 / 600 / 600 / 600	96.00	0.348	1.196	1.187
	38					
F2	1	3000	97.00	0.055	1.276	1.164
(0.477)	2	1000 / 2000	97.00	0.147	1.273	1.168
	2	1500 / 1500	97.00	0.150	1.272	1.162
	3	750 / 750 / 1500	97.00	0.236	1.269	1.167
	3	1000/ 1000 / 1000	96.50	0.226	1.270	1.171
	4	750 / 750 / 750 / 750	96.00	0.320	1.266	1.168
	5	600 / 600 / 600 / 600 / 600	95.50	0.384	1.264	1.171

In Table B, we present the results using the proposed meta-analysis framework applied to heavily skewed raw data. Specifically, following the approach of Cao et al. (2014), we used a non-normally distributed outcome Y in all simulation settings. Here, ε_2 was simulated from a $\chi^2(2)$ distribution and scaled to replicate the heavily skewed

outcome. The results indicate that the coverage and bias patterns are similar to those observed with normally distributed outcomes, demonstrating excellent performance.

Table B. Simulation results for scenarios (D1) with skewed data. CP refers to the empirical coverage probability of 95% confidence intervals based on 200 replications. Q refers to the number of studies. SE refers to the average asymptotic standard error. SD refers to the empirical standard deviation of replicated estimations.

Model	Scenario	Q	N	**CP** %	**Bias** $\times 10^{-2}$	**SE** $\times 10^{-2}$	**SD** $\times 10^{-2}$
Fixed Effects	D1 - Independent (0.322)	1	3000	96.0	-0.039	1.405	1.323
		2	1000 / 2000	96.5	-0.016	1.403	1.324
		2	1500 / 1500	96.0	-0.014	1.403	1.323
		3	750 / 750 / 1500	96.5	-0.002	1.402	1.330
		3	1000/ 1000 / 1000	96.5	0.000	1.403	1.328
		4	750 / 750 / 750 / 750	97.0	0.021	1.401	1.332
		5	600 / 600 / 600 / 600 / 600	96.5	0.067	1.400	1.332
Fixed Effects	D1 - Correlated (0.369)	1	3000	97.5	-0.079	1.399	1.279
		2	1000 / 2000	96.5	0.022	1.398	1.432
		2	1500 / 1500	91.5	-0.135	1.404	1.542
		3	750 / 750 / 1500	96.0	-0.087	1.394	1.325
		3	1000/ 1000 / 1000	91.5	-0.392	1.405	1.553
		4	750 / 750 / 750 / 750	94.5	-0.264	1.413	1.492
		5	600 / 600 / 600 / 600 / 600	93.0	-0.405	1.404	1.455
Random Effects (DerSimonian-Laird)	D1 - Independent (0.380)	5	2400	84.0	-1.686	12.584	11.712
		8	1500	88.5	-2.148	10.790	8.815
		10	1200	92.5	-2.129	10.049	7.563
		16	750	94.0	-1.879	8.136	6.408
		20	600	94.0	-1.740	7.450	6.096
Random Effects (Median-unbiased PM)	D1 - Independent (0.380)	5	2400	90.0	-1.679	12.357	11.697
		8	1500	92.0	-2.142	9.748	8.805
		10	1200	95.0	-2.124	8.752	7.556
		16	750	94.5	-1.875	6.865	6.405
		20	600	91.0	-1.737	6.142	6.099
Random Effects (DerSimonian-Laird)	D1 - Correlated (0.380)	5	2400	85.5	-1.704	12.526	11.679
		8	1500	88.5	-2.320	10.668	8.837
		10	1200	92.5	-2.171	9.967	7.512
		16	750	92.5	-2.043	8.140	6.408
		20	600	94.0	-1.827	7.510	6.101
Random Effects (Median-unbiased PM)	D1 - Correlated (0.380)	5	2400	90.5	-1.694	12.396	11.660
		8	1500	93.5	-2.310	9.786	8.822
		10	1200	94.0	-2.161	8.803	7.498
		16	750	94.0	-2.038	6.902	6.404
		20	600	92.0	-1.825	6.187	6.103

DETAILS AND SUPPLEMENT OF THE APPLICATIONS

We applied the FDR-adjusted p-value to filter out selected genes not associated with the exposures, setting the FDR cutoff point at 0.2. We conducted pathway enrichment analysis using the Database for Annotation, Visualization and Integrated Discovery (DAVID) (Dennis Jr et al., 2003) to evaluate the significance of those mediating genes enriched in specific pathways.

Table C presents the meta-analysis results by sample size-weighted Stouffer's combination of p-values (Willer et al., 2010) for pathways identified for systolic BP from the Kyoto Encyclopedia of Genes and Genomes (KEGG) (Kanehisa and Goto, 2000), which were ranked by the meta-analysis p-value. Table D lists the pathways identified for HDL-C.

Table C. The Top 10 significant pathways and p-value identified for systolic blood pressure. Micro_GEN3 refers to the FHS Third Generation cohort with microarray gene expression profiling. Micro_OFF refers to the FHS Offspring cohort with microarray gene expression profiling. RNA_GEN3 refers to the FHS Third Generation cohort with RNAseq gene expression profiling. RNA_OFF refers to the FHS Offspring cohort with RNAseq gene expression profiling. Overall refers to the overall p-value using the sample size based METAL.

Pathway	Count	Genes	Micro_GEN3	Micro_OFF	RNA_GEN3	RNA_OFF	MESA	Overall
hsa04810:Regulation of actin cytoskeleton	3	CXCL12, NCKAP1L, ITGAL	0.536	0.758	0.145	1.000	0.025	0.030
hsa03013:Nucleocytoplasmic transport	3	NUP214, RNPS1, IPO5	0.181	0.328	1.000	1.000	1.000	0.296
hsa04814:Motor proteins	3	KIF1C, KIF17, ACTG1	0.754	0.624	0.274	1.000	1.000	0.344
hsa01100:Metabolic pathways	5	UGT8, RRM2, ATIC, HYAL4, PGM2L1	0.998	0.626	0.932	0.858	0.335	0.448
hsa04141:Protein processing in endoplasmic reticulum	5	GANAB, RPN1, DERL1, STT3A, SEC24C	1.000	0.113	1.000	1.000	1.000	0.456
hsa05132:Salmonella infection	6	TUBB6, ARHGEF26, NCKAP1L, VPS39, S100A10, DNM2	1.000	0.715	0.709	1.000	0.456	0.487
hsa04120:Ubiquitin mediated proteolysis	2	HUWE1, BIRC6	0.642	0.790	0.533	1.000	1.000	0.511
hsa05203:Viral carcinogenesis	4	RASA2, UBR4, CHD4, CCR4	0.773	0.382	1.000	1.000	1.000	0.591
hsa04380:Osteoclast differentiation	3	FCGR3A, ATP2A2, FYN	1.000	0.432	1.000	1.000	1.000	0.712
hsa05200:Pathways in cancer	6	LAMA5, CXCL12, LAMA1, LAMC3, LAMA3, COL4A6	0.901	0.691	0.946	1.000	0.879	0.731

Table D. The Top 10 significant pathways and p-value identified for HDL-C. Micro_GEN3 refers to the FHS Third Generation cohort with microarray gene expression profiling. Micro_OFF refers to the FHS Offspring cohort with microarray gene expression profiling. RNA_GEN3 refers to the FHS Third Generation cohort with RNAseq gene expression profiling. RNA_OFF refers to the FHS Offspring cohort with RNAseq gene expression profiling. Overall refers to the overall p-value using the sample size based METAL.

Pathway	Count	Genes	Micro_GEN3	Micro_OFF	RNA_GEN3	RNA_OFF	MESA	Overall
hsa04144:Endocytosis	6	GRK2, GRK6, VPS26B, TGFBR2, DNM2, HLA-E	0.262	0.556	0.113	0.147	0.311	0.011
hsa04072:Phospholipase D signaling pathway	2	DNM2, PIK3R5	0.457	0.669	0.096	0.008	1.000	0.020
hsa04010:MAPK signaling pathway	5	MAP3K2, CACNG8, JUN, RASGRF2, NF1	0.203	0.182	0.658	0.214	1.000	0.066
hsa04810:Regulation of actin cytoskeleton	3	CXCL12, NCKAP1L, ITGAL	0.693	0.821	0.821	0.007	0.276	0.070
hsa04910:Insulin signaling pathway	2	SREBF1, EXOC7	0.764	0.269	0.080	0.521	1.000	0.073
hsa05012:Parkinson disease	3	PSMC5, GPR37, SLC25A31	0.941	0.046	0.865	0.052	1.000	0.085
hsa04151:PI3K-Akt signaling pathway	6	LAMA5, FLT1, LAMA1, LAMC3, LAMA3, COL4A6	0.317	0.750	0.125	0.574	0.809	0.090
hsa01100:Metabolic pathways	6	PIGT, GPAT4, ME1, UQCR11, GAPDH, ATP6V0C	0.756	0.351	0.830	0.055	0.559	0.109
hsa04630:JAK-STAT signaling pathway	2	IL21R, IL5RA	0.518	0.711	0.034	1.000	1.000	0.115
hsa04150:mTOR signaling pathway	2	STK11, FZD6	1.000	0.689	0.028	0.568	1.000	0.122

Figure A presents the application results using 5 cohorts for the systolic BP and HDL-C. Given the observed lack of heterogeneity between cohorts for the systolic BP outcome ($I^2 = 0\%$, $p = 0.6851$), the fixed-effects model

similarly concluded that 3.6% (95% CI = (2.6%, 4.7%)) of the variance in systolic BP could be explained by age through gene expression. For HDL-C, the observed heterogeneity between cohorts for the HDL-C outcome (I^2 = 57.02%, $p = 0.0539$) is moderate even though it is not statistically significant. The DL estimator indicated that 8.1% (95% CI = (5.9%, 10.3%)) of the variation could be explained by sex through gene expression, with the MPM estimator providing a nearly identical estimate of 8.1% (95% CI = (6.0%, 10.2%)). We also performed the fixed-effects model and concluded that 8.8% (95% CI = (7.5%, 10.1%)) of the variation could be explained by sex through gene expression.

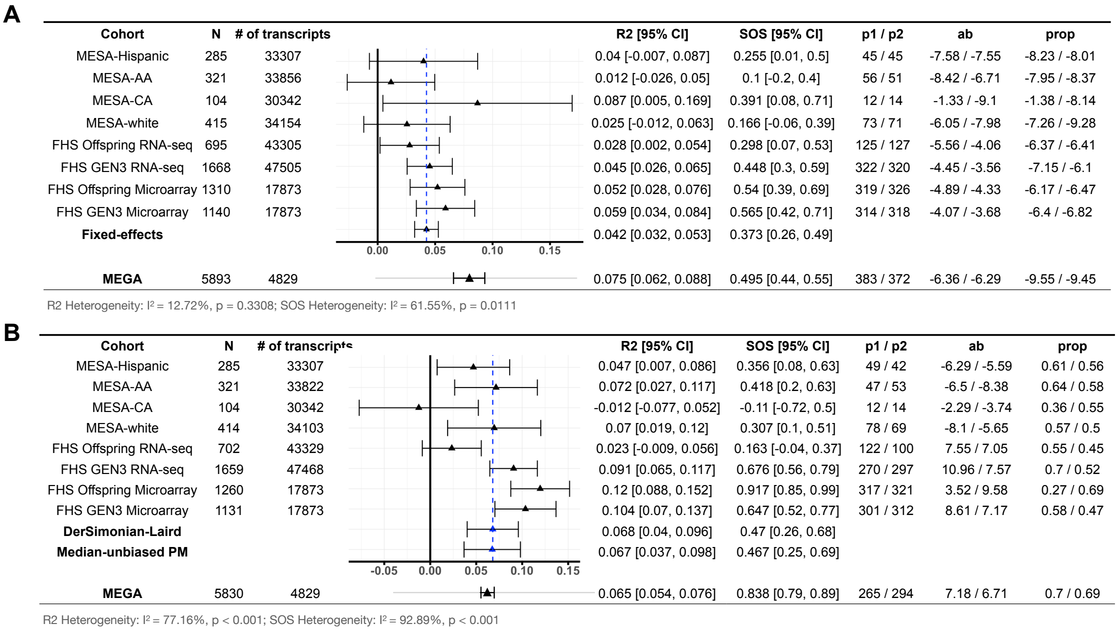

Fig. A. Meta-analysis results of mediation effect of gene expression between age and systolic BP using the CF-OLS in 8 different cohorts from the NHLBI TOPMed program. N refers to the sample size. # of transcripts refers to the number of genes measured from the gene expression profiling. $p1/p2$ refers to the number of transcripts selected in the first and second subsample, respectively. R2 refers to the total mediation effect R^2_{Med}. CI refers to the confidence interval. ab refers to the product measure in the first and second subsample. prop refers to the proportion measure in the first and second subsample.

Table E shows the number of overlapping transcripts across different studies using various technologies. Notably, the FHS microarray contains 17,873 genes, and after combining with the FHS RNA-seq studies, over 14,730 genes remain. The different RNA-seq studies all have more than 44,000 transcripts within the study. However, when MESA is combined with the FHS studies, only 5,370 common genes are left, representing a significant reduction. This highlights that using our proposed meta-analysis method is more reliable and reasonable than pooling all studies together for inference.

Mediation analysis with single mediator

In Figure B and Figure C, we present the Venn diagrams showing the selected genes across five cohorts for the systolic BP and HDL outcomes. We chose the gene ATP-binding cassette transporters G1 (*ABCG1*) as the mediator, as it was shared across four studies for the HDL outcome, to perform the R^2-based mediation analysis and compare the results with mean-based measures such as the product and proportion measures.

Table E. Number of overlapped transcripts between different studies.

**Study**	FHS Offspring-RNAseq	FHS Gen3-RNAseq	FHS-Microarray	MESA-RNAseq
FHS Offspring-RNAseq	48384	47519	14730	34679
FHS Gen3-RNAseq	/	51928	14844	38038
FHS-Microarray	/	/	17873	5370
MESA-RNAseq	/	/	/	44069

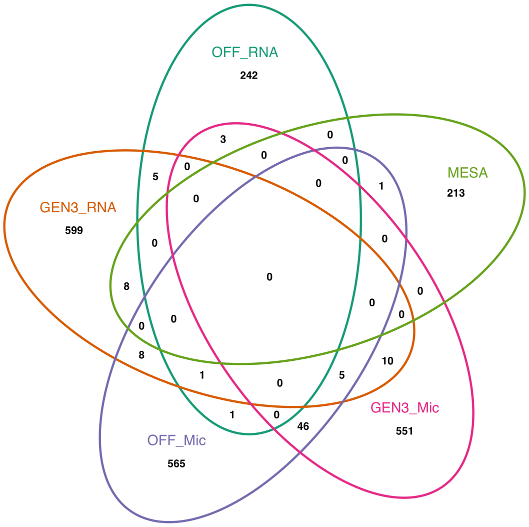

Fig. B. Venn diagram of selected genes in 5 cohorts with Systolic BP outcome.

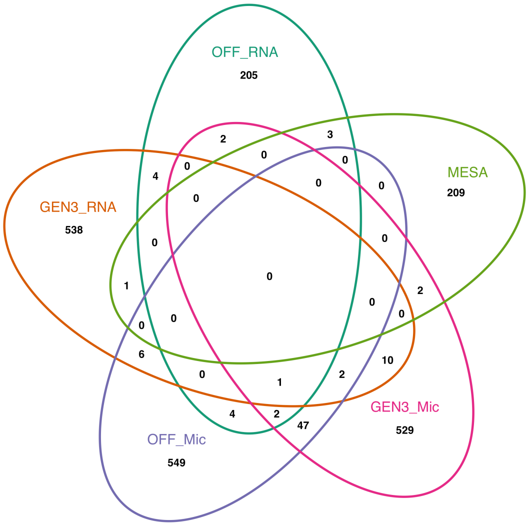

Fig. C. Venn diagram of selected genes in 5 cohorts with HDL outcome.

Table F presents the results from the single mediator meta-analysis, showing that the R^2 values are heterogeneous, whereas the SOS values are homogeneous. For the product measure ab and the proportion measure $prop$, we used 200 bootstrap resampling-based standard errors to conduct the meta-analysis. Both measures were found to be homogeneous based on our tests, which justified the use of a fixed-effects model. Of note, *ABCG1* has been shown to play an important role in regulating HDL in a sex-dependent manner (Wang et al., 2004; Jin et al., 2023).

Table F. Meta-analysis results of mediation effect of single gene ABCG1 expression between sex and HDL using the CF-OLS in 5 different cohorts from the NHLBI TOPMed program. N refers to the sample size. # of transcripts refers to the number of genes measured from the gene expression profiling. R2 refers to the total mediation effect R^2_{Med}. CI refers to the confidence interval. se refers to the standard error. ab refers to the product measure. prop refers to the proportion measure.

Cohort	N	Technology	R2 [95% CI]	R2 - se	SOS [95% CI]	SOS - se	ab	ab - se	prop	prop - se
FHS-Offspring	702	RNA-seq	0.006 [-0.004, 0.015]	0.005	0.038 [-0.03, 0.10]	0.033	0.005	0.008	0.014	0.020
FHS-Generation 3	1659	RNA-seq	0.007 [0.001, 0.013]	0.003	0.052 [0.00, 0.10]	0.025	-0.012	0.005	-0.031	0.015
FHS-Offspring	1260	Microarray	0.003 [-0.005, 0.010]	0.004	0.020 [-0.04, 0.08]	0.030	-0.005	0.006	-0.013	0.017
FHS-Generation 3	1131	Microarray	0.002 [-0.003, 0.007]	0.003	0.014 [-0.02, 0.04]	0.016	-0.004	0.004	-0.011	0.010
MESA	1124	RNA-seq	0.001 [-0.006, 0.008]	0.004	0.004 [-0.03, 0.04]	0.017	0.000	0.005	-0.001	0.012
**Fixed Effects**			**0.0035 [0.0005, 0.0064]**		**0.0190 [0.0003, 0.0377]**		**-0.0042[-0.0087, 0.0003]**		**-0.0137 [-0.0253, -0.0020]**	

R2 Heterogeneity: I2 = 0%, p = 0.6882; SOS Heterogeneity: I2 = 0.74%, p = 0.5617; ab Heterogeneity: I2 = 14.36%, p = 0.228; ab Heterogeneity: I2 = 9.10%, p = 0.4928.

REFERENCES

Y. Cao, P. Wei, M. Bailey, J. S. Kauwe, T. J. Maxwell, and A. D. N. Initiative. A versatile omnibus test for detecting mean and variance heterogeneity. *Genetic epidemiology*, 38(1):51–59, 2014.

G. Dennis Jr, B. Sherman, D. Hosack, J. Yang, W. Gao, H. Lane, et al. David: Database for annotation, visualization, and integrated discovery genome biol 4 (9): R60–r60. 11. *Find this article online*, 2003.

J. Jin, X. Zhao, C. Zhu, and et al. Hypomethylation of abcg1 in peripheral blood as a potential marker for the detection of coronary heart disease. *Clin Epigenet*, 15:120, 2023.

M. Kanehisa and S. Goto. Kegg: kyoto encyclopedia of genes and genomes. *Nucleic acids research*, 28(1):27–30, 2000.

N. Wang, D. Lan, W. Chen, F. Matsuura, and A. Tall. Atp-binding cassette transporters g1 and g4 mediate cellular cholesterol efflux to high-density lipoproteins. *Proc Natl Acad Sci U S A.*, 101(26):9774–9779, 2004.

C. J. Willer, Y. Li, and G. R. Abecasis. Metal: fast and efficient meta-analysis of genomewide association scans. *Bioinformatics*, 26(17):2190–2191, 2010.
